# The new cardioprotector Monohydroxyethylrutoside protects against doxorubicin-induced inflammatory effects *in vitro*

**DOI:** 10.1038/sj.bjc.6601022

**Published:** 2003-07-15

**Authors:** M A I Abou El Hassan, H M W Verheul, A S Jorna, C Schalkwijk, J van Bezu, W J F van der Vijgh, A Bast

**Affiliations:** 1 Department of Medical Oncology, Free University Medical Center, PO Box 7057, 1007 MB Amsterdam, The Netherlands; 2 Department of Clinical Chemistry, Free University Medical Center, PO Box 7057, 1007 MB Amsterdam, The Netherlands; 3 Department of Pharmacology and Toxicology, University of Maastricht, PO Box 616, 6200 MD Maastricht, The Netherlands

**Keywords:** doxorubicin, monoHER, HUVECs, MTT, neutrophil adhesion

## Abstract

Besides its cardiotoxic effect, doxorubicin also elicits inflammatory effects *in vivo*. 7-Monohydroxyethylrutoside (monoHER) has recently been used as a protector against doxorubicin-induced cardiotoxicity *in vivo*. It is not known yet whether monoHER can also protect against doxorubicin-induced inflammatory effects. The aim of the present study was (1) to illustrate the inflammatory effects of doxorubicin *in vitro* and (2) to evaluate a possibly protective effect of monoHER. In order to demonstrate the inflammatory effects of doxorubicin and the possible protection of monoHER, proliferating human umbilical cord vascular endothelial cells (HUVECs) were incubated with different concentrations of doxorubicin ranging from 12.5 to 600 nM with(out) 200 *μ*M monoHER. Resting (confluent) HUVECs were incubated with (0.5–25 *μ*M) doxorubicin with(out) monoHER (0.2–1.2 mM) and the viability of endothelial cells and their propensity to adhere to neutrophils were measured 24 h after treatment. The localisation of adhered neutrophils was determined with immunofluorescence microscopy. To further characterise the mechanism of doxorubicin-induced neutrophil adhesion, the expression of the HUVECs surface adhesion molecules was determined after doxorubicin treatment. Doxorubicin decreased the viability and proliferation capacity of HUVECs in a concentration-dependent manner. The proliferating HUVECs were much more sensitive to doxorubicin (IC_50_=60.0±20.8 nM) than resting cells (LC_50_=4.0±0.3 *μ*M). Doxorubicin also increased the adhesion of neutrophils reaching a plateau value at a doxorubicin concentration of ⩾0.4*μ*M (*P*=0.0113). The induced neutrophil adhesion was accompanied by overexpression of VCAM and E-selectin but not ICAM. Although monoHER did not reverse the effect of doxorubicin on the proliferation of endothelial cells, it significantly protected resting HUVECs against the cytotoxic effect of doxorubicin (⩽25 *μ*M, *P*<0.0015). In addition, monoHER completely protected against the stimulatory effect of doxorubicin on neutrophil adhesion, and inhibited the doxorubin-induced expression of VCAM and E-selectin on the surface of treated HUVECs. This study illustrates that monoHER, which protects against doxorubicin's cardiotoxic effect, can also protect against doxorubicin-induced inflammatory effects. These data prompt further investigation about the possible link between doxorubicin-induced inflammatory effects and its cardiotoxicity *in vivo*.

Doxorubicin is a well-known chemotherapeutic agent used against a wide range of human malignancies. The development of acute and chronic cardiotoxicity (Schwartz *et al*, 1993), however, limits the clinical use of doxorubicin. The mechanism of doxorubicin-induced cardiotoxicity is still not fully understood. The prevailing hypothesis, however, suggests that the cardiotoxicity results from the formation of reactive oxygen species (ROS) (e.g. hydroxyl (OH^.^) and superoxide anion (O^−.^_2_) radicals) (Olson and Mushlin, 1990; Takacs *et al*, 1992; Goeptar *et al*, 1994). Reactive oxygen species affect heart tissue specifically because of its relatively low antioxidants content (e.g. SOD and catalase) and the relative abundance of mitochondria (Takacs *et al*, 1992).

In addition to its cardiotoxic effects, doxorubicin elicits also inflammatory effects as illustrated by the occurrence of phlebitis (Hecker, 1990) and acute inflammatory reactions in the eyelid of different laboratory animals (McLoon and Wirtschafter, 1997). Doxorubicin also causes inflammatory reactions in the vicinity of heart tissue where it was found to increase the incidence of thrombus formation in the atrium of mice (Fujihira *et al*, 1993).

Recently, we have shown that 7-monohydroxyethylrutoside (monoHER) could protect against doxorubicin-induced cardiotoxicity in the mouse (Van Acker *et al*, 2000; Van Acker *et al*, 1997). MonoHER cardioprotection is believed to result from the protection against doxorubicin-induced free radicals as a good antioxidant and metal ion chelating agent (Haenen *et al*, 1993; Van Acker *et al*, 1993). Since the mechanism of monoHER is not fully established, it is also important to study the effect of monoHER on the inflammatory effects of doxorubicin.

Accordingly, the aim of this study was to establish a model showing the inflammatory effects of doxorubicin *in vitro*. Using this model, the effect of doxorubicin on the viability and proliferating capacity of human vascular endothelial Cells (HUVECs) was studied. Furthermore, the effect of doxorubicin on the neutrophil adhesion of HUVECs was studied. In the mean time, the expression of several adhesion molecules on the surface of doxorubicin-treated HUVECs was also studied. The study was completed by investigating the protection obtained by monoHER against the inflammatory effects of doxorubicin in that model.

## MATERIALS AND METHODS

### Chemicals

7-monohydroxyethylrutoside was kindly provided by Novartis Consumer Health (Nyon, Switzerland). Doxorubicin HCl was purchased from Pharmacia Upjohn BV (Woerden, The Netherlands). Potassium chloride, sodium chloride, ammonium chloride, EDTA (disodium salt), potassium bicarbonate and dimethyl sulphoxide (DMSO) were purchased from Merck (Amsterdam, The Netherlands), bovine serum albumin, trypsin, 3-(4,5-dimethylthiazol-2-yl)-2,5-diphenyltetrazolium bromide (MTT) and phenyl methyl sulphonyl chloride (PMSF) from Sigma-Aldrich Chemie (Zwijndrecht, The Netherlands), D-glucose and nonidet P40 (NP40) from Fluka Biochemika (Buchs, Switzerland), human serum from CLB (Amsterdam, The Netherlands) and M199, fetal calf serum (FCS) and HBSS (without calcium) from Life Technologies (Breda, The Netherlands). Calcein AM was from Molecular Probes Europe BV (Leiden, The Netherlands), Ficoll from Pharmacia Biotech AB (Upssala, Sweden) and tumour necrosis factor *α* (TNF*α*), L-glutamine, fibronectin (human plasma), heparin and HEPES from ICN Biomedicals (Aurora, OH). Endothelial cell growth factor (ECGF) was extracted from bovine hypothalamus as previously mentioned (Maciag *et al*, 1979).

### Human vascular endothelial cells isolation and culture

Human vascular endothelial cells were isolated as previously described (Verheul *et al*, 2000). In brief, the umbilical cord vein was filled with trypsin/EDTA, after being washed with warm PBS, and incubated at 37°C for 20 min. The dissociated cells were collected by washing the vein with PBS and the cell suspension was centrifuged at 241 **g** for 7 min. The cell pellet was quickly resuspended in culture medium (M199 containing 10% human serum, 10% FCS, 5 U ml^−1^ heparin, 200 IE ml^−1^ penicillin and 200 *μ*g streptomycin, 0.29  mg ml^−1^ L-glutamine and 50 *μ*g ml^−1^ ECGF). Endothelial cells passage 2 (P2), plated in fibronectin-precoated 96-well flat-bottom microtitre plate (Costar, Badhoevedorp, The Netherlands), was used during the whole study.

### Proliferation assay (IC_50_)

Human vascular endothelial cells were plated on precoated 96-well plate with a density of 3000 cells/well. Cells were incubated with different concentrations of doxorubicin (12.5−600 nM, prepared in culture medium) with(out) (200 *μ*M) monoHER. Dimethyl sulphoxide was used as a solubiliser for monoHER with a final concentration of 0.1%, which did not show any antiproliferative effect. Cells were washed (3 ×) with warm medium 24 h after treatment and were left to grow for another 48 h. Thereafter, the MTT assay was performed according to Mosmann (1983) with minor modifications. The percentage growth (proliferation) was calculated by dividing the increase in the cell count (after the time of the experiment, i.e. 3 days) of the treated cells by the increase of the cell count of the untreated cells (which was taken as 100% proliferation). The decrease in the proliferation capacity (as percentage of control) was plotted as a function of the doxorubicin concentration. The IC_50_ of doxorubicin with(out) monoHER was estimated from the plot.

### Cytotoxicity assay (LC_50_)

In the cytotoxicity studies confluent HUVECs were used. The LC_50_ was determined by treating confluent cells with different concentrations of doxorubicin (0.5–25 *μ*M) with(out) monoHER (0.2–1.2 mM). The viability was measured 24 h after treatment using the MTT assay. Dimethyl sulphoxide, used as a solubilizer for monoHER, reached a final concentration of 0.5%, which hardly decreased (5%) the viability of confluent HUVECs. The percentage viability (taking the control as 100% viability) was plotted as a function of doxorubicin or monoHER concentration. The LC_50_ of doxorubicin with(out) monoHER was estimated.

### Isolation and preparation of calcein-labelled human neutrophils

Isolation of neutrophils was performed as previously reported (Schaefer *et al*, 1998) with a slight modification. In short, 24 ml of venous blood was drawn, by venipuncture, from healthy donors into vacutainer tubes (Becton Dickinson, Plymouth, UK) containing 0.11 M sodium citrate and centrifuged at 1500 g for 5 min. The buffy coat was carefully layered over a Ficoll solution and was centrifuged at 540 g for 20 min at room temperature. Neutrophils were isolated from the red pellet after the complete lysis of the erythrocytes (with lysis buffer containing 0.15 M NH_4_Cl, 1 mM KHCO_3_ and 0.1 M Na_2_EDTA) at 0°C followed by centrifugation at 380 **g** for 5 min at 4°C. After counting, the cell density was adjusted to 2.5 × 10^6^ cell/ml in M199 medium. Neutrophils were labelled by incubation with 2.5 *μ*M of the florescent indicator calcein AM in the dark for 15 min at 37°C.

### Neutrophil adhesion assay

Confluent HUVECs were incubated for 24 h with different concentrations (range 0.2–1.0 *μ*M) of doxorubicin, prepared in M199 containing 10% human serum, alone or in combination with 1 mM of monoHER (dissolved in 0.5% DMSO-containing medium). Dimethyl sulphoxide did not affect the adhesion of neutrophils to HUVECs *in vitro*. After stimulation, HUVECs were washed with prewarmed M199 (2X). 100 *μ*l of the calcein-labelled neutrophils was added per well and left to adhere for 1 h at 37°C. Subsequently, the unbound neutrophils were thoroughly washed away with warm PBS (3X). The fluorescent dye was released by lysis of the bound neutrophils with 50 *μ*l of lysis buffer containing 1% NP40, 150 mM NaCl, 10 mM Tris-HCl (pH 7.5), 5 mM EDTA and 1 mM PMSF. After 10 min of shaking, the fluorescence was measured with a spectrafluor multiplate reader TECAN (Salzburg, Austria) with excitation and emission filters of 492 and 535 nm, respectively. The percentage adhesion (taking the control as zero percent adhesion) was plotted as a function of doxorubicin concentration.

For the validation of the procedure, TNFα (1000 U ml^−1^) was used as a positive control. As previously reported (Clark *et al*, 1997) TNFα stimulation caused an increase in neutrophil adhesion of HUVECs with a factor of 2.5–4 compared to the blank.

### Immunofluorescence microscopy

To localise neutrophil adhesion to doxorubicin-stimulated HUVECs, bound neutrophils were not lysed but fixed with 4% paraformaldehyde for 15 min. After washing, cells were incubated with 10% FCS and 0.02 M glycine in PBS for 30 min followed by incubation with the primary antibodies CD 15 FITC (1 : 40 dilution) (Becton Dickinson, Leiden, The Netherlands) and biotinylated ulex europaeus agglutinin 1 (1 : 100 dilution) (Vector Laboratories, Inc., Burlingame, CA, USA) for the neutrophils and the endothelial cells, respectively. After washing with PBS (3X), streptavidin-Texas red (Vector Lab., Inc.), as fluorescent labelled secondary antibody, was added (1 : 100 dilution) for the detection of the biotinylated lectin. After washing, the wells were carefully dried and mounted with Vectashield mounting medium for fluorescence (H1200, Vector Lab. Inc.). Stained wells were examined with an, LEICA confocal microscope showing neutrophils in green and HUVECs in red. A rarely appearing yellow colour represented areas of coincident labelling with both antibodies.

### Expression of cell surface adhesion molecules

Confluent HUVECs, cultured in M199 containing 10% human serum, were incubated for 24 h with different concentrations (range 0.08–1.0 *μ*M) of doxorubicin alone or in combination with 1 mM of monoHER (dissolved in 0.13% DMSO-containing medium). Dimethyl sulphoxide did not affect the expression of the cell surface VCAM, ICAM or E-selectin. Under these conditions, the positive control, that is, cells treated with TNFα (10 U ml^−1^), induced between three- and five-fold increase in the expression of all the adhesion molecules.

The expression of cell-bound VCAM-1, ICAM-1 and E-selectin was measured as described earlier (Carlos *et al*, 1990). The colour intensity was measured at a wavelength of 450 nm. Experiments were performed in triplicate. The results were expressed as percentage of the control (taking the control as zero control), which was plotted as a function of doxorubicin concentration.

### Statistical analysis

The results are expressed as the mean value of three different days (±s.e.m.). The value of each day represents the mean value of three wells. The cells used on each day were obtained from a separate umbilical cord. The overall effect of doxorubicin with(out) monoHER was evaluated with ANOVA. When the ANOVA indicated significant effect, the contrasts (differences) of the effect of each concentration from the blank were investigated. For monoHER protection, the contrast between monoHER effect at each doxorubicin concentration (viz. the difference between doxorubicin+monoHER and doxorubicin alone) and monoHER alone (viz. taking the change due to monoHER alone into account) was also investigated.

The comparison of the IC_50_ or the LC_50_ of doxorubicin alone or with monoHER was made using student's *t*-test.

## RESULTS

### Antiproliferative effect of doxorubicin (IC_50_)

**Figure 1:**
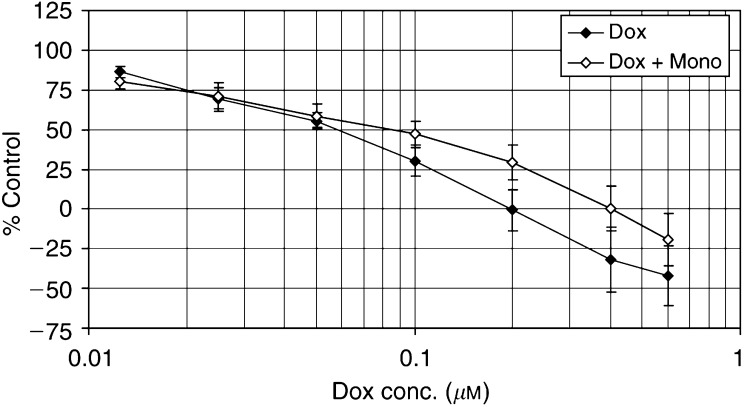
The effect of doxorubicin with(out) monoHER on the proliferation of HUVECs 24 h after treatment compared to control. Values are mean (*n*=3)±s.e.m.

The antiproliferative effect of doxorubicin on HUVECs is shown in Figure 1

. Doxorubicin decreased the proliferation of endothelial cells in a concentration-dependent manner and the IC_50_ was 60±21 nM after 24 h. Coincubation with 200 *μ*M monoHER did not significantly change the IC_50_ of doxorubicin (93±54 nM).

### Cytotoxic effect of doxorubicin (LC_50_)

In this assay, confluent HUVECs were used to exclude proliferation of the endothelial cells. Confluent HUVECs were 65-fold more resistant to doxorubicin compared to proliferating cells and the LC_50_ was 4.0±0.3 *μ*M.

**Figure 2:**
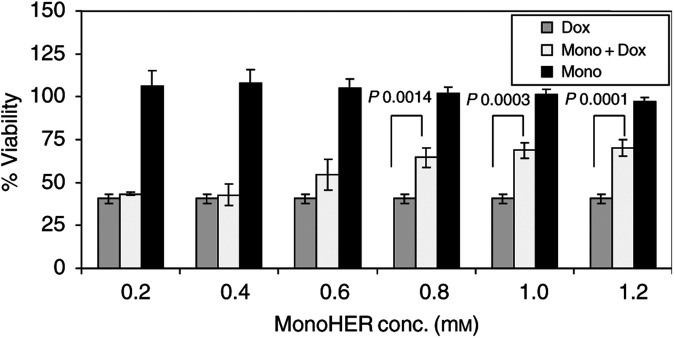
The viability of endothelial cells 24 h after treatment with 5 *μ*M doxorubicin with(out) different concentrations of monoHER. Values are mean (*n*=3)+s.e.m. The *P* values, indicating monoHER concentrations that significantly protected against the damaging effect of (5 *μ*M) doxorubicin, were calculated with ANOVA.

The protective effect of different concentrations of monoHER against 5 *μ*M doxorubicin (which is almost equal to the LC_50_ of doxorubicin) is shown in Figure 2

. Forty percent of HUVECs remained viable after incubation with 5 *μ*M doxorubicin for 24 h. 7-Monohydroxyethlrutoside (0.2–1.2 mM) alone did not affect the viability of confluent HUVECs. Human vascular endothelial cells were significantly protected against the cytotoxic effect of doxorubicin at monoHER concentrations ⩾0.8 mM (*P*=0.0014) compared to doxorubicin alone. Maximum protection (about 30% of the viability) was attained when a monoHER concentration of ⩾1 mM (*P*=0.0003) was used.

Figure 3

**Figure 3:**
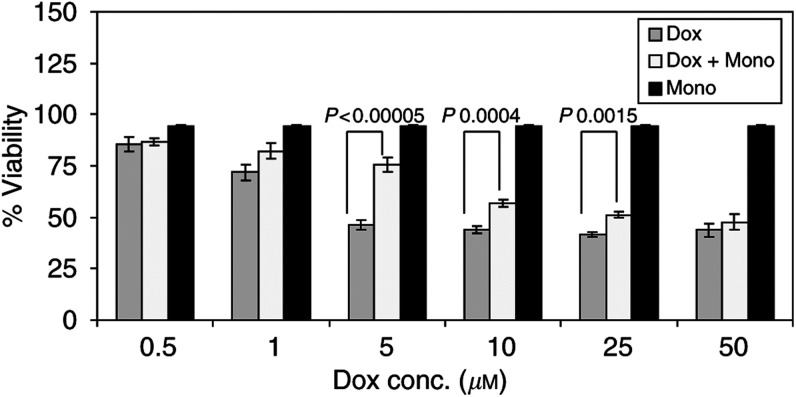
The viability of endothelial cells 24 h after treatment with different concentrations of doxorubicin with(out) 1 mM monoHER. Values are mean (*n*=3)+s.e.m. The *P* values, which indicate the significant protection of monoHER against the damaging effect of different concentrations of doxorubicin, were calculated with ANOVA.

shows the viability of confluent HUVECs 24 h after exposure to different concentrations of doxorubicin with(out) 1 mM monoHER. Doxorubicin significantly decreased the viability of confluent HUVECs in a concentration-dependent manner (*P*<0.00005). Coincubation with 1 mM monoHER protected endothelial cells against doxorubicin and the LC_50_ of doxorubicin significantly increased from 4±0.3 to 40±10 *μ*M (*P*<0.05). 7-Monohydroxyethykrutoside protection was dependent on the doxorubicin concentration: maximum protection was obtained when the doxorubicin concentration was 5 *μ*M (*P*<0.00005) and significant protection could still be obtained when doxorubicin concentrations were ⩽25 *μ*M (*P*=0.0015).

### Immunofluorescence microscopy

**Figure 4:**
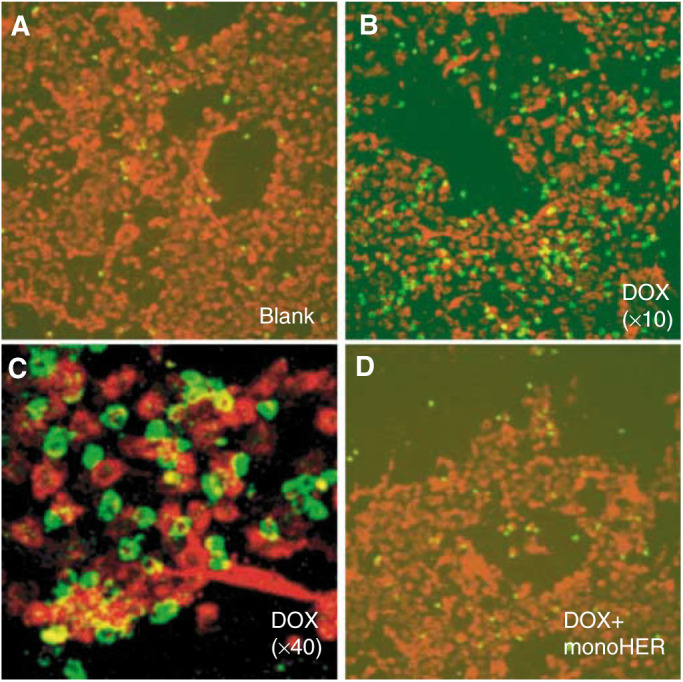
Immunofluorescence microscopy of neutrophil (in green) adhesion after stimulation with doxorubicin (0.6* μ*M) with (out) 1 mM monoHER compared to unstimulated HUVECs (in red).

Immunofluorescence microscopy was used for the localization of adhering neutrophils upon stimulation with doxorubicin with(out) monoHER. As a result of the cellular damage induced by doxorubicin, concentrations of ⩽1 *μ*M were used in order to reduce the nonspecific adhesion of neutrophils (i.e. to the fibronectin-coated bottom). Figure 4

shows the adhesion of neutrophils (in green) to HUVECs (in red) after stimulation with 0.6 *μ*M doxorubicin with(out) 1 mM monoHER in comparison to unstimulated cells (blank). Few neutrophils were bound to unstimulated HUVECs. Neutrophil adhesion increased by stimulation of HUVECs with 0.6 *μ*M doxorubicin. Neutrophils were specifically bound to the endothelial cells as shown in Figure 4C (× 40). Coincubation with monoHER completely reverted doxorubicin-induced neutrophil adhesion back to basal levels (Figure 4D).

### Adhesion of neutrophils to stimulated HUVECs

Figure 5

**Figure 5:**
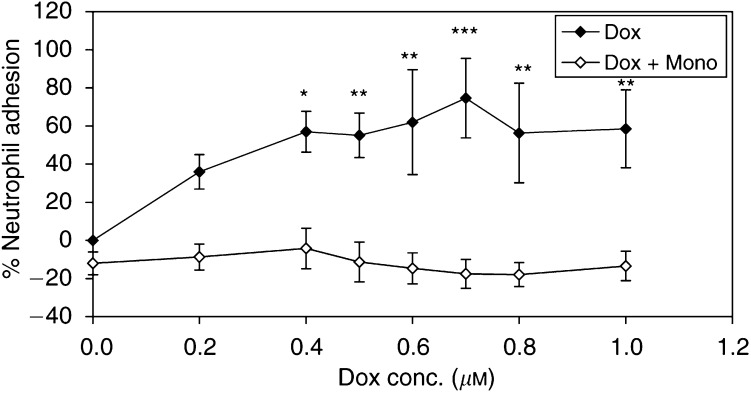
The adhesion of neutrophils to HUVECs (expressed as percentage of the control) was observed 24 h after stimulation with doxorubicin alone or in combination with (1 mM) monoHER. Values are mean (*n*=3)±s.e.m. The *P*-values (^*^<0.02, ^**^<0.01, ^***^<0.001), which indicate significant protection of monoHER against doxorubicin-induced neutrophil adhesion of HUVECs, were calculated with ANOVA.

shows the percentage adhesion of neutrophils to confluent HUVECs 24 h after stimulation with different concentrations of doxorubicin (0.2–1.0 *μ*M) alone or in combination with (1 mM) monoHER. Doxorubicin stimulation of endothelial cells resulted in a significant increase in the percentage neutrophil adhesion, as detected by the increase of the fluoresence, compared to the control. This increase was concentration dependent and reached a plateau at doxorubicin concentrations of ⩾0.4 *μ*M (*P*<0.02). 7-Monohydroxyethylrutoside alone did not affect the adhesion of neutrophils to HUVECs. Simultaneous incubation with (1 mM) monoHER reverted doxorubicin-induced adhesion to the basal levels (Figure 5).

### Overexpression of cell bound adhesion molecules

**Figure 6:**
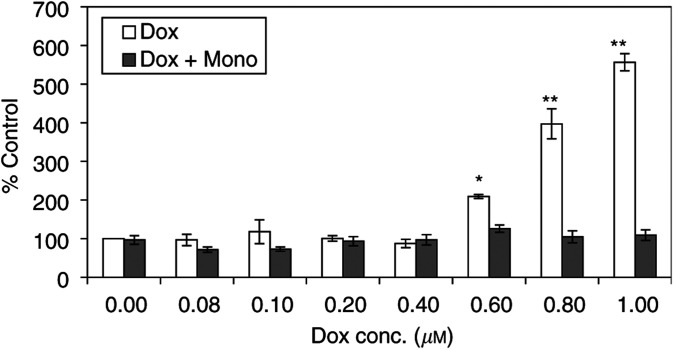
The overexpression of VCAM 24 h after stimulation with doxorubicin with(out) 1 mM monoHER. Values are mean (*n*=3)±s.e.m. The *P* values (^*^< 0.002, ^**^< 0.001), which indicate significant increase in the expression of VCAM, were calculated with ANOVA.

**Figure 7:**
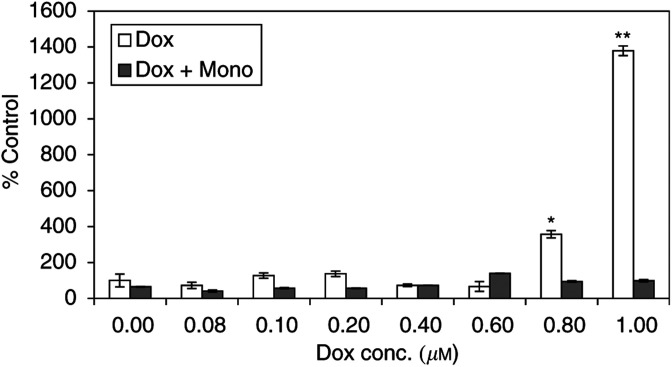
The overexpression of E-selectin 24 h after stimulation with doxorubicin with(out) 1 mM monoHER. Values are mean (*n*=3)±s.e.m. The *P* values (^*^< 0.001, ^**^< 0.0005), which indicate significant increase in the expression of VCAM, were calculated with ANOVA.

Figure 6 and Figure 7

show the expression of VCAM and E-selectin on the surface of HUVECs, as a percentage from the control, 24 h after stimulation with different concentrations of doxorubicin with(out) 1 mM monoHER. Both VCAM and E-selectin expression increased significantly (*P*<0.002) after treatment with doxorubicin concentrations >0.6 and >0.8 *μ*M, respectively. The expression level of ICAM did not change after the same treatment. The combined treatment with 1 mM monoHER reverted doxorubicin-stimulated overexpression of VCAM and E-selectin to the basal levels.

## DISCUSSION

7-Monohydroxyethylrutoside was recently used in the protection against doxorubicin-induced cardiotoxicity *in vivo* (Van Acker *et al*, 1997, 2000). The mechanism of protection is not fully understood. The prevailing hypothesis is scavenging of free radicals produced by doxorubicin and chelation of iron ions (Haenen *et al*, 1993). Besides cardiotoxic properties, doxorubicin has also inflammatory effects. It was therefore thought important to study the role of monoHER against these inflammatory effects.

Thusfar the inflammatory effects of doxorubicin did not have much attention. The inflammatory effects of doxorubicin were not only found in the vasculature as phlebitis (Hecker, 1990) or as skin inflammation after local treatment in the eye lid of several laboratory animals (McLoon and Wirtschafter, 1997) but also in the vicinity of the heart tissue. An increase in neutrophil adhesion and thrombus formation has been found in the atrium of 75% of mice repeatedly injected with 4 mg kg^−1^ doxorubicin (Fujihira *et al*, 1993). It is remarkable that the authors found associated damage in the cardiomyocytes and the presence of interstitial inflammatory cell infiltration. These findings suggest that the inflammatory effect of doxorubicin may be directly or indirectly related to its cardiotoxicity.

In the present study, we have illustrated the inflammatory effects of doxorubicin *in vitro*. Doxorubicin reduced the viability of resting endothelial cells with concentrations comparable to the concentrations attained in the plasma of human and laboratory animals (Mross *et al*, 1990; Van der Vijgh *et al*, 1990). In addition to the direct damage to the vascular endothelial cells, doxorubicin also induced neutrophil adhesion of vascular endothelial cells *in vitro*.

In order to study the process of neutrophil adhesion to doxorubicin-stimulated HUVECs, the expression of surface-bound adhesion molecules was studied. The present study shows that doxorubicin-induced neutrophil adhesion to stimulated HUVECs was mediated via the overexpression of VCAM and E-selectin but not via ICAM. Furthermore, it also shows that the overexpression of VCAM occurs after stimulation with a lower concentration of doxorubicin (0.6 *μ*M) than that for E-selectin (0.8 *μ*M doxorubicin).

Besides the inflammatory effect, the present study has also illustrated that doxorubicin affects proliferating endothelial cells much more markedly (IC_50_=60 nM) than resting (confluent) cells (LC_50_=4 *μ*M). Since proliferating endothelial cells are involved in the neovascularisation (angiogenesis), this may indicate that a low dose of doxorubicin may have an antiangiogenic effect without affecting the vascular endothelium (resting endothelial cells with a low turnover rate; Klohs and Hamby, 1999). This finding is supported by Browder *et al* (2000), who have recently shown the antiangiogenic effect of low-dose chemotherapy.

The present study is the first to show the anti-inflammatory properties of the flavonoid monoHER. The addition of monoHER significantly protected against doxorubicin damaging effect on resting HUVECs *in vitro*. 7-Monohydroxyethylrutoside also completely inhibited doxorubicin-induced neutrophil adhesion of HUVECs *in vitro*. This protection may be mediated by the observed inhibition of doxorubicin-induced VCAM and E-selectin overexpression. On the other hand, monoHER did not protect against the antiproliferative effect of doxorubicin.

The anti-inflammatory effect of the flavonoid monoHER could be expected. Different flavonoids have an anti-inflammatory effect, which plays a role in their therapeutic effect on allergy, asthma, viral infection and gastric ulcers (Gerritsen *et al*, 1995; Havsteen, 1983). The good antioxidant and metal ion chelating properties of monoHER (Haenen *et al*, 1993; Van Acker *et al*, 1993) play a role in the protection of endothelial cells against the direct damage of doxorubicin. These properties enable monoHER to protect against doxorubicin-induced oxygen radicals, which induce lysis of the endothelial cells and increase the vascular permeability and reactivity (Clark *et al*, 1997). In addition, monoHER is one of the constituents of Venoruton®, which is used in the treatment of chronic venous insufficiency by decreasing vascular permeability and protecting vascular endothelial cells (Havsteen, 1983). The protection of monoHER did not interfere with the antiproliferative effect of doxorubicin. This is in line with our previous work where monoHER protected against doxorubicin-induced cardiotoxicity without interfering with its antiproliferative effect both *in vitro* and *in vivo* (Van Acker *et al*, 1997).

The present *in vitro* study together with several publications, pose an important question about a possible link between doxorubicin-induced inflammatory effects and its cardiotoxicity. Our *in vitro* data show that doxorubicin affected both the viability and neutrophil adhesion of endothelial cells with clinically achievable concentrations. Others have also shown that doxorubicin has vascular effects *in vivo*. Doxorubicin was found to disrupt the balance of several agents involved in the regulation of vasodilatation, vasoconstriction and neutrophil adhesion *in vivo*, for example, endothelin-1 (ET-1), histamine (Decorti *et al*, 1989,^1997^; Duffy *et al*, 1999) and nitric oxide (NO) (Bristow *et al*, 1980), leading to an imbalance in the physiology of the vasculature especially after the long-term use of doxorubicin. Besides the vascular imbalance, doxorubicin damage to endothelial cells could cause endothelial dysfunction, which is known to induce myocardial ischaemia, aggravate acute coronary syndromes and accelerate progression of chronic artery disease (Britten and Schachinger, 1998). These observations suggest the occurrence of cardiovascular ailment after doxorubicin administration, which pinpoints to a link between the inflammatory effects of doxorubicin and its induced cardiotoxicity. As a result of the potential clinical applicability, it is important to confirm the link between the inflammatory effects of doxorubicin and its cardiotoxicity in an *in vivo* model and to study the extent by which these inflammatory effects participate in the development of the heart disease. This can be studied by investigating whether the anti-inflammatory effects of monoHER and other anti-inflammatory agents could partially protect against doxorubicin-induced cardiotoxicity *in vivo*.

## CONCLUSION

Doxorubicin, in clinically relevant concentrations, showed inflammatory effects *in vitro* (reduced the viability of HUVECs and increased their propensity for neutrophil adhesion via VCAM and E-selectin but not via ICAM). Proliferating endothelial cells were much more sensitive to doxorubicin damage than resting endothelial cells. 7-monohydroxyethylrutoside protected against these inflammatory effects without interfering with the antiproliferative effect of doxorubicin. The possibility of a relation between doxorubicin-induced-inflammatory effects and its cardiotoxicity should be further investigated *in vivo*.
